# Esterification of *p*-Coumaric Acid Improves the Control over Melanoma Cell Growth

**DOI:** 10.3390/biomedicines11010196

**Published:** 2023-01-12

**Authors:** Joana I. Carmo-Martins, Michelangelo B. Gonzatti, Marina T. Varela, Maria Eduarda P. Sousa, Lucas V. S. Costa, Elaine Guadelupe Rodrigues, João Paulo S. Fernandes, Alexandre C. Keller

**Affiliations:** 1 Department of Microbiology, Immunology, and Parasitology, Division of Immunology, Escola Paulista de Medicina, Universidade Federal de São Paulo, *campus* São Paulo, São Paulo 04023-062, Brazil; 2 Department of Pharmaceutical Sciences, Institute of Environmental, Chemical and Pharmaceutical Sciences, Universidade Federal de São Paulo, *campus* Diadema, Diadema 09913-030, Brazil; 3 Department of Microbiology, Immunology, and Parasitology, Division of Cell Biology, Escola Paulista de Medicina, Universidade Federal de São Paulo, *campus* São Paulo, São Paulo 04023-062, Brazil

**Keywords:** melanoma, B16-F10, *p*-coumaric acid, tumor, cell growth

## Abstract

Previous studies reported that *p*-coumaric acid modulates melanoma growth. Because the esterification of *p*-coumaric acid (*p*-CA) enhanced its activity as an antimelanogenic agent, we aimed to determine the antitumor potential of two derivatives, the ethyl and butyl esters, against the murine B16-F10 and the human SK-MEL-25 melanoma cells. Cell viability was determined in vitro by the lactate dehydrogenase release and violet crystal absorption assays. The cell proliferation rate and cell cycle behavior were determined by the colony formation assay and flow cytometry analysis. Although *p*-CA, at the concentration of 1 mM, failed to exert a significant antitumor activity, the ethyl and butyl ester derivatives caused substantial tumor cell death at doses < 1 mM. Despite a reduction in their direct cytotoxicity at minor doses, both products controlled the melanoma growth by arresting the cell cycle at the G0/G1 (B16-F10) or S/G2 (SK-MEL-25). Furthermore, the in vivo experiments showed that the butyl ester derivative suppressed the lung B16-F10 burden, compared to the *p*-CA-treated mice. Thus, the esterification of *p*-coumaric acid improved the control over the proliferation of murine and human melanoma cells and can be considered an approach for designing novel anticancer agents.

## 1. Introduction

Cutaneous melanoma is one of the most common skin cancers and the most aggressive dermatologic malignancy, being a leading cause of morbidity and mortality worldwide [1]. Although surgical excision at the early stages of the melanoma development provides a good prognosis (especially at stage I), treatment at more advanced stages requires systemic therapies, such as radio- or chemotherapy, targeted therapy, or immunotherapy [2]. Unfortunately, the risk of tumor recurrence, undesired collateral effects, and the high cost of specific treatments, remain obstacles to a better prognosis for many melanoma patients. Alternatively, natural compounds commonly found in plants exhibit several pharmacological activities, including anticancer properties, and represent a potential alternative to improve cancer management. In this sense, the study by Hu et al. demonstrated that *p*-coumaric acid (*p*-CA), a phenolic phenylpropanoid acid widely found in mushrooms, grains, fruits, and vegetables, controls the in vitro growth of melanoma cells [3]. According to this study, *p*-CA induced, in vitro, both apoptosis and cell cycle arrest in the human A375 (2.5–3.5 mM) and murine B16 (3.0–4.0 mM) melanoma cell lines [3].

In addition to the direct effect on the melanoma viability, *p*-CA also acts as an antimelanogenic agent in melanocytes, by competing with tyrosine for the active site of tyrosinase [4]. In a previous study, we demonstrated that the esterification of *p*-CA increased both its lipophilicity and antimelanogenic activity, indicating that *p*-CA derivatives exhibit a higher biological activity than their parental acid [5]. Therefore, we aimed to determine the antitumor potential of two selected compounds against the melanoma cells: the ethyl *p*-coumarate (**1**) and the *n*-butyl *p*-coumarate (**2**) (Figure S1).

Our in vitro results show that compounds **1** and **2** are more efficient to induce tumor cell death and control the cell proliferation at lower doses than *p*-CA. Furthermore, an in vivo metastatic assay revealed that compound **2**, but not *p*-CA, significantly decreased the lung tumor load. Therefore, our data support the notion that the *p*-CA esterification improves its antitumor properties and opens new perspectives in cancer management. 

## 2. Materials and Methods

### 2.1. p-Coumaric acid and Ester Derivatives

*p*-Coumaric acid (*p*-CA) was obtained from Sigma-Aldrich (Darmstadt, DE). The ethyl *p*-coumarate (**1**) and the *n*-butyl *p*-coumarate (**2**) were synthesized following the classic Fisher esterification, as previously described by our group and maintained in a 100 mM stock solution in DMSO (Sigma-Aldrich, Saint Louis, MO, USA) [5]. 

### 2.2. Animals

Male, wild type C57BL/6J mice, aged 8–10 weeks were obtained from CEDEME (Centro de Desenvolvimento de Modelos Experimentais para Medicina e Biologia). All animals were allocated in a specific pathogen-free environment with filtered water and standard solid food ad libitum. The animal study protocol was approved by the local ethical committee (Unifesp- CEUA nº 3646251021, São Paulo, SP, Brazil). All animal procedures were performed according to the Federal Law 11.794 (2008), The ARRIVE guidelines and the Guide for the Care and Use of Laboratory Animals of the Brazilian National Council of Animal Experimentation (CONCEA).

### 2.3. Cell Lines and Cell Culture

Mouse melanoma B16-F10 and human melanoma SK-MEL-25 cell lines were obtained from Banco de Células do Rio de Janeiro (BCRJ, Rio de Janeiro, RJ, Brazil). Both cell lines were maintained in DMEM (Gibco-ThermoFisher, Grand Island, NY, USA) supplemented with 10% FBS (Gibco-ThermoFisher, Grand Island, NY, USA), 100 U/mL penicillin, 100 μg/mL streptomycin (Gibco-ThermoFisher, Grand Island, NY, USA), and 1 mM sodium pyruvate (Gibco-ThermoFisher, Grand Island, NY, USA), at a humid atmosphere of 5% CO_2_ at 37 °C.

### 2.4. Cell Cytotoxicity and Viability Assay

The B16-F10 and SK-MEL-25 cells (3 × 10^3^/well) were seeded in 96-well flat bottom plate and left overnight for complete adherence. Then, the cells were treated with *p*-CA or compounds **1** or **2** at concentrations between 1 to 0.06 mM for 4 h or 24 h. To determine the cell cytotoxicity, the supernatant was collected to address the release of lactate dehydrogenase (LDH) from the damaged cells, according to the adapted manufacturer’s protocol (Quibasa-Bioclin, Belo Horizonte, MG, Brazil). Following the removal of the supernatant, the cells were gently washed with PBS, stained/fixed with crystal violet solution (0.5% in acetic acid 30%) for 15 min, washed with tap water and left to dry at room temperature. Then, the crystal violet was dissolved with methanol and the optical density was determined at 570 nm (OD_570_). The crystal violet solution stains live adhered cells; thus, the cell viability was obtained as: %_viability_ = Sample OD_570_.100/Control OD_570_, where Sample represents the OD after treatment and Control represents the average OD of the non-treated cells set as 100% of viable cells (n = 5) [6]. In addition, the induction of apoptosis was analyzed by a flow cytometry assay. Briefly, the cells were incubated for 2 h with *p*-CA, compound **1** or **2**, as indicated in the Figures S2 and S3. Following the wash, the cells were incubated with an annexin V solution (eBioscience, San Diego, CA, USA) to stain the apoptotic cells, followed by the incubation with a LIVE/DEAD™ Fixable Aqua Dead Cell Stain Kit (ThermoFischer, Eugene, OR, USA), to discriminate the dead cells using a FACS Canto-II (BD Biosciences, San Diego, CA, USA) flow cytometry system. The apoptotic *versus* dead cells analysis was carried out using FlowJo version 10.2 (BD Biosciences, Ashland, OR, USA).

### 2.5. Cell Proliferation Assay

The B16-F10 and SK-MEL-25 cells were stained with 5 μM carboxyfluorescein succinimidyl ester (CFSE; ThermoFisher, Eugene, OR, USA) for 20 min at 37 °C. Then, the cells were washed with a complete medium, and seeded (10^4^/well) in a 24-well flat bottom plate. Following the adhesion, the cells were treated with 0.1 mM of *p*-CA or compounds **1** or **2** for 72 h. The CFSE fluorescence intensity peaks were analyzed over this period by flow cytometry, using a FACS Canto-II (BD Biosciences, USA) flow cytometry system [7]. The cell proliferation analysis was carried out using FlowJo version 10.2 (BD Biosciences, Ashland, OR, USA).

### 2.6. Cell Cycle Assay

The B16-F10 and SK-MEL-25 cells (5 × 10^5^/well) were seeded in a 6-well flat bottom plate and left overnight for complete adherence. Then, the cells were treated with 0.1 mM of *p*-CA or compounds **1** or **2** for 24 h. Following this period, the cells were collected, washed with PBS, fixed in cold 70% ethanol on ice for 2 h. Following the washing with PBS, the cells were incubated for 30 min with the DAPI staining solution (PBS 0.1% Triton X-100 and 1 μg/mL 4,6-diamino-2-phenyl-indole), in the dark, at room temperature. Following the washing, the cells were analyzed for DNA content using a FACS Canto-II (BD Biosciences, USA) flow cytometry system [8]. In addition, some cells were treated with a fixation and permeabilization buffer for staining with anti-Ki-67 (anti-mouse: eBioscience, San Diego, CA, USA/anti-human: BD Biosciences, San Diego, CA, USA). The cell cycle and the expression of the Ki-67 analysis were carried out using FlowJo version 10.2 (BD Biosciences, Ashland, OR, USA).

### 2.7. Colony Formation Assay

The B16-F10 and SK-MEL-25 cells (150/well) were seeded in a 6-well flat bottom plate and left overnight for complete adherence. Then, the cells were treated with 0.1 mM of *p*-CA or compounds **1** or **2** for 72 h. Following this period, the cells were maintained in a fresh medium for an additional 4 days. Then, the cells were fixed with 4% paraformaldehyde for 15 min, stained with the crystal violet solution for 15 min, washed with tap water, and left to dry at room temperature for later observation of the colony formation [9].

### 2.8. In Vitro Splenocyte Stimulation

The splenocytes were obtained after the tissue dissociation with a 70 μM cell strainer, followed by erythrocyte lysis with an ACK solution (0.15 M NH_4_Cl, 10 mM KHCO_3_, 0.1 mM Na_2_ EDTA). The total splenic cells were cultured with *p*-CA or compounds **1** or **2** (0.1 mM) for 6 h. Then, the cells were incubated with LIVE/DEAD Fixable Aqua Dead Stain Kit (ThermoFischer, Eugene, OR, USA) in the presence of Fc blocking (anti-CD16/32), washed, and labeled with anti-CD3 (APC-Cy7), anti-CD8 (PE-Cy7), anti-NK1.1 (FITC), and anti-CD69 (eFluor^TM^450) (All antibodies are from eBioscience, San Diego, CA, USA). The live single cells were analyzed for the CD69 expression (MFI) in the CD8 T lymphocytes (CD3^+^CD8^+^) and the NK cells (CD3^−^NK1.1^+^), using a FACS Canto-II (BD Biosciences, USA) flow cytometry system [8]. The cell analysis was carried out using FlowJo version 10.2. (BD Biosciences, Ashland, OR, USA). 

### 2.9. In Vivo Treatment and the Metastasis Model

The C57BL/6J mice were injected intraperitoneally (i.p.) with a saline solution containing *p*-CA or compound **2** (100 mg/Kg) for 5 consecutive days. the control animals received the corresponding DMSO concentration. At day 3 of the treatment, 2 × 10^5^ B16-F10 melanoma cells were inoculated intravenously (i.v.), through the tail vein, and the number of tumor nodules was assessed 15 days later.

### 2.10. Statistical Analysis

For multiple comparisons, we executed a two-way or one-way ANOVA, followed by Tukey’s multiple comparison test. All statistical analyses were performed using GraphPad Prism software version 7 (San Diego, CA, USA). Data represent the compilation of two individual experiments (n = 5/group) mean ± standard error of the mean (SEM). Values of *p* < 0.05 were considered statistically significant.

## 3. Results

### 3.1. p-Coumaric Acid Esterification Improves the Cytotoxicity against the B16-F10 Melanoma Cells

**Figure 1:**
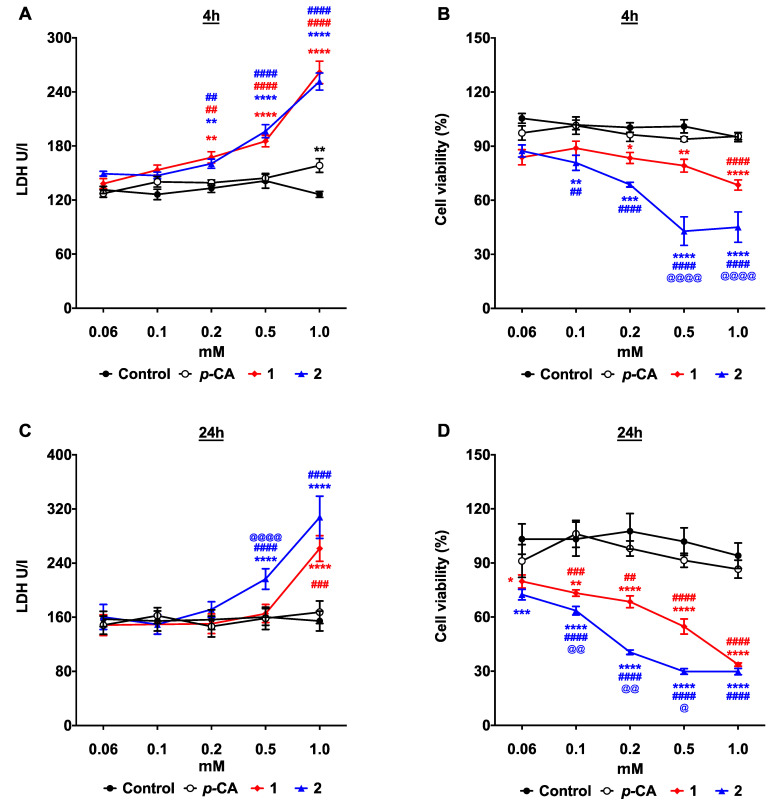
*p*-Coumaric acid esterification improves the cytotoxicity against the B16-F10 melanoma cells. Murine B16-F10 melanoma cells were incubated with different concentrations of *p*-coumaric acid (*p*-CA, white circles), ethyl *p*-coumarate (**1**, red lozenges), or *n*-butyl *p*-coumarate (**2**, blue triangles) for 4 h or 24 h. Control cells were incubated with the equivalent concentrations of DMSO. (**A**,**C**) LDH quantification in culture supernatants; (**B**,**D**) frequency of viable cells. * *p* < 0.05, ** *p* < 0.01, *** *p* < 0.001, **** *p* < 0.0001 compounds **1** or **2** vs. Control; ## *p* < 0.01, ### *p* < 0.001, #### *p* < 0.0001 compounds **1** or **2** vs. *p*-CA; @ *p* < 0.05, @@ *p* < 0.005, @@@@ *p* < 0.0001 compound **2** vs. **1**.

Although, after 4 h of treatment, the *p*-CA group exhibited a significant LDH release at the concentration of 1 mM, there is no significant alteration in the frequency of the viable cells, compared to the non-treated (control) cells. Thus, this data indicates that at this dose, *p*-CA did not exert an important cytotoxic effect on the B16-F10 melanoma cells (Figure 1A,B, respectively). Indeed, there is no significant cytotoxic effect on the B16-F10 cells, even after 24 h of incubation with *p*-CA, in any concentration (Figure 1C,D). In contrast, both ester derivatives, **1** and **2**, exerted a significant cytotoxic effect against B16-F10 at the 4 h and 24 h time points when the concentration was >0.1 mM. Although, after 4 h, both compounds induced a significant LDH release in a concentration ranging from 0.2 to 1 mM, compound **2** exerted a greater cytotoxic effect than compound **1** at the concentration of 0.5 and 1.0 mM (Figure 1B). For notice, the most effective antitumor effect against the B16-F10 cells is more evident after 24 h of treatment. At this time point, although the LDH release was only significant for both compounds **1** and **2** at the concentrations of 0.5 and 1.0 mM (Figure 1C), the cell viability assay shows a decrease in the frequency of the viable cells starting from the concentration of 0.06 mM (Figure 1D). The idea that compounds **1** and **2** have a higher cytotoxic potential was corroborated by a flow cytometry assay. As soon as two hours after incubation were completed, compounds **1** and **2**, but not *p*-CA, induce a significant increase in the frequency of dead cells, with a more pronounced effect observed for compound **2** (Figure S2). Therefore, these data support the idea that the *p*-CA esterification improves its cytotoxic effect against the B16-F10 melanoma cells.

### 3.2. Ethyl p-Coumarate (***1***) and n-Butyl p-Coumarate (***2***) Inhibit the B16-F10 Cell Proliferation by Inducing the Cell Cycle Arrest at the G0/G1 Phase

**Figure 2:**
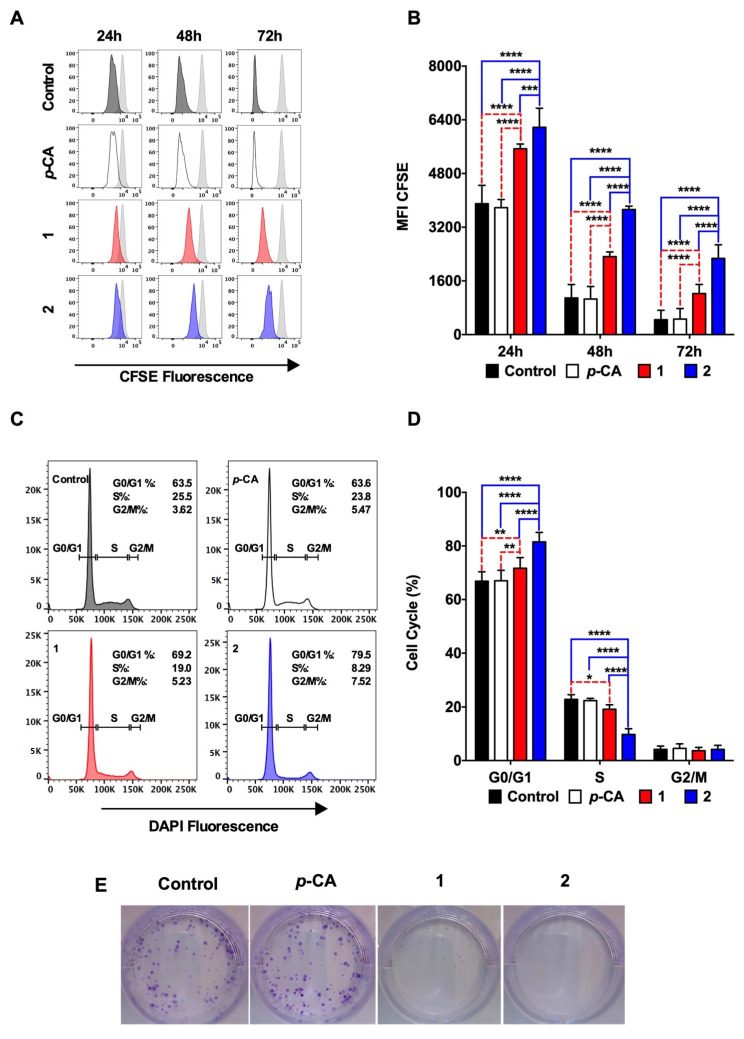
Ethyl *p*-coumarate (**1**) and n-butyl *p*-coumarate (**2**) inhibits the B16-F10 cell proliferation and induces the cell cycle arrest at the G0/G1 phase. Murine B16-F10 melanoma cells were incubated with *p*-coumaric acid (*p*-CA, white), ethyl *p*-coumarate (**1**, red) or n-butyl *p*-coumarate (**2**, blue) at 0.1 mM. (**A**,**B**) cells were cultured for 24, 48, and 72 h in the presence of compounds and stained with CFSE, to determine the cell proliferation over 72 h; (**C**,**D**) cells were cultured for 24 h in the presence of compounds and stained with DAPI to analyze the cell cycle profile; (**E**) cells were cultured for 72 h in the presence of compounds and cultured for an additional four days in a fresh medium, to determine their capacity to form cell colonies. Figure represents a single sample from n = 3/group of the two independent experiments. Control groups were incubated with the concentration of DMSO equivalent to those at 0.1 mM of the compounds. * *p* < 0.05, ** *p* < 0.01, *** *p* < 0.001, **** *p* < 0.0001.

The antiproliferative capacity of the ester derivatives was determined by incubating the B16-F10 cells with the compounds **1** and **2** at 0.1 mM for 72 h, and the intensity of the CFSE fluorescence was analyzed at 24 h, 48 h, and 72 h. Figure 2A,B show that compounds **1** and **2** diminished the CFSE decay over time, indicating that both *p*-coumarate derivatives reduced the B16-F10 cell proliferation. In concordance, the cell cycle analysis revealed a cell arrest at the G0/G1 phase (Figure 2C,D). Noteworthy, the effects of compound **2** on the B16-F10 proliferation were more pronounced than those observed in the cells treated with compound **1**.

A colony formation assay was performed to determine the antiproliferative effect over time. Following 72 h of incubation with *p*-CA or compounds **1** or **2** (0.1 mM), the tumor cells were cultured for 4 additional days in a fresh medium, and the formation of cell colonies was observed in both the control and the *p*-CA cells. In contrast, compounds **1** or **2**-treated cells failed to form significant cell colonies (Figure 2E).

### 3.3. Ethyl p-Coumarate (***1***) and n-Butyl p-Coumarate (***2***) Exert Cytotoxic and Antiproliferative Effects against the SK-MEL-25 Melanoma Cells

**Figure 3:**
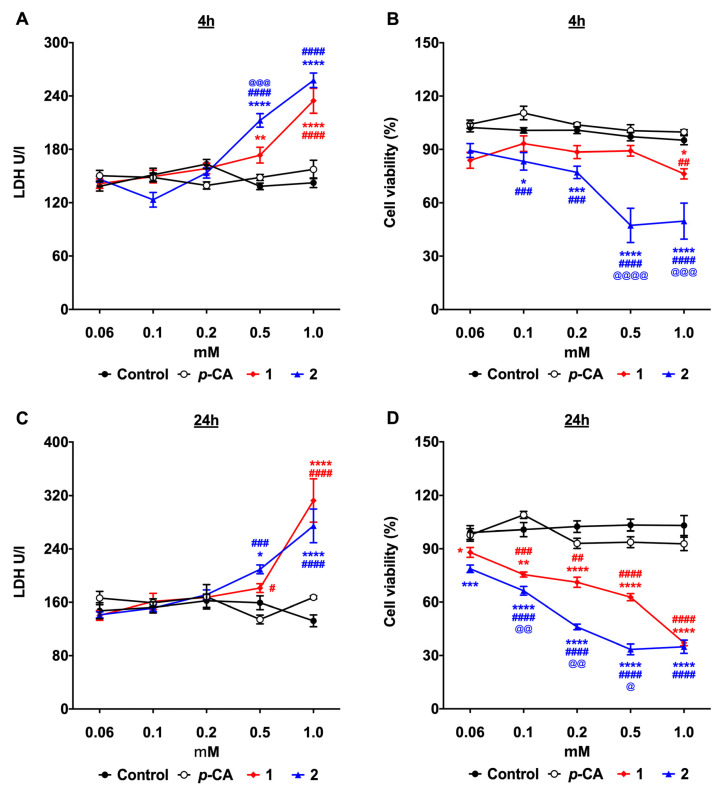
*p*-Coumaric acid esterification improves the cytotoxicity against the SK-MEL-25 melanoma cells. Human SK-MEL-25 melanoma cells were incubated with different concentrations of *p*-coumaric acid (*p*-CA, white circles), ethyl *p*-coumarate (**1**, red lozenges), or *n*-butyl *p*-coumarate (**2**, blue triangles) for 4 h or 24 h. Control cells were incubated with the equivalent concentrations of DMSO. (**A**,**C**) LDH quantification in the culture supernatants; (**B**,**D**) frequency of the viable cells. * *p* < 0.05, ** *p* < 0.01, *** *p* < 0.001, **** *p* < 0.0001 compounds **1** or **2** vs. control; # *p* < 0.05, ## *p* < 0.01, ### *p* < 0.001, #### *p* < 0.0001 compounds **1** or **2** vs. *p*-CA; @ *p* < 0.05, @@ *p* < 0.01, @@@ *p* < 0.001, @@@@ *p* < 0.0001 compound **2** vs. **1**.

The antitumor potential of compounds **1** and **2** on SK-MEL-25, a human melanoma cell line, was assessed using the experimental conditions previously described for the B16-F10 cells. In contrast to *p*-CA, which had no cytotoxic effect on the SK-MEL-25 cells, compounds **1** and **2**, at the concentration of 0.5 and 1.0 mM, induced the LDH release at 4 h and 24 h (Figure 3A,C). As previously observed for the murine B16-F10 melanoma cells, despite a significant impact on the cell viability at 4 h of incubation, their antitumor effect was more evident after 24 h (Figure 3B,D). Likewise observed with the B16-F10 cells, compound **2** exhibited the highest antitumor activity. Indeed, the flow cytometry analysis revealed that after 2 h of incubation, compound **2**, at the concentration of 0.5 mM, can already induce a significant increase in the frequency of dead cells, compared to compound **1** and *p*-CA (Figure S3).

**Figure 4:**
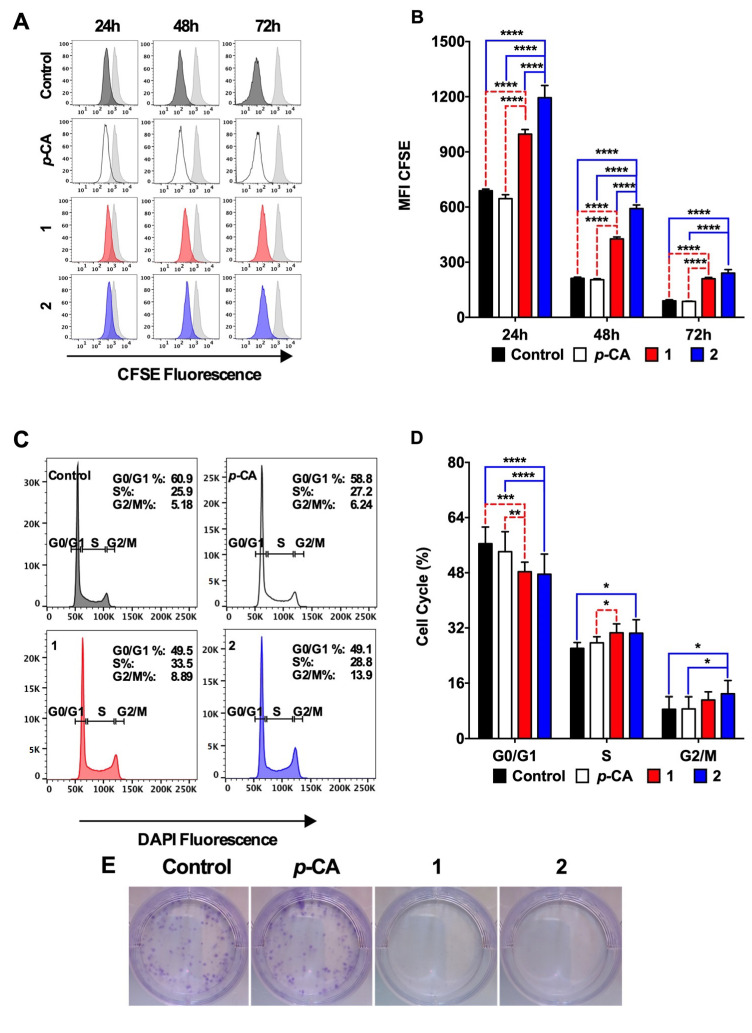
Ethyl *p*-coumarate (**1**) and *n*-Butyl *p*-coumarate (**2**) inhibits the SK-MEL-25 cell proliferation and induces the cell cycle arrest at the S and G2/M phases. Human SK-MEL-25 melanoma cells were incubated with *p*-coumaric acid (*p*-CA, white), ethyl *p*-coumarate (**1**, red) or *n*-butyl *p*-coumarate (**2**, blue) at 0.1 mM. (**A**,**B**) cells were cultured for 24, 48, and 72 h in the presence of the compounds and stained with CFSE to determine the cell proliferation over 72 h; (**C**,**D**) cells were cultured for 24 h in the presence of the compounds and stained with DAPI to analyze the cell cycle profile; (**E**) cells were cultured for 72 h in the presence of compounds and cultured for an additional seven days in a fresh medium to determine their capacity to form cell colonies. This figure represents a single sample from n = 3/group of the two independent experiments. Control cells were incubated with the concentration of DMSO equivalent to those at 0.1 mM of the compounds. * *p* < 0.05, ** *p* < 0.01; *** *p* < 0.001, **** *p* < 0.0001.

Finally, we used a flow cytometry assay to determine the antiproliferative capacity of compounds **1** and **2**. The CFSE analysis showed that both compounds efficiently inhibited the SK-MEL-25 proliferation over 72 h of culture (Figure 4A,B). The cell cycle analysis revealed that, differently from that observed in the B16-F10 cells, compounds **1** and **2** induced a cell cycle arrest at the S phase, with some cells also arrested at the G2/M phase in the presence of compound **2** (Figure 4C,D). The colony formation assay corroborated the antiproliferative effect on SK-MEL-25 by compounds **1** and **2** (Figure 4E).

### 3.4. N-Butyl p-Coumarate (***2***) Controls the In Vivo Tumor Metastasis

**Figure 5:**
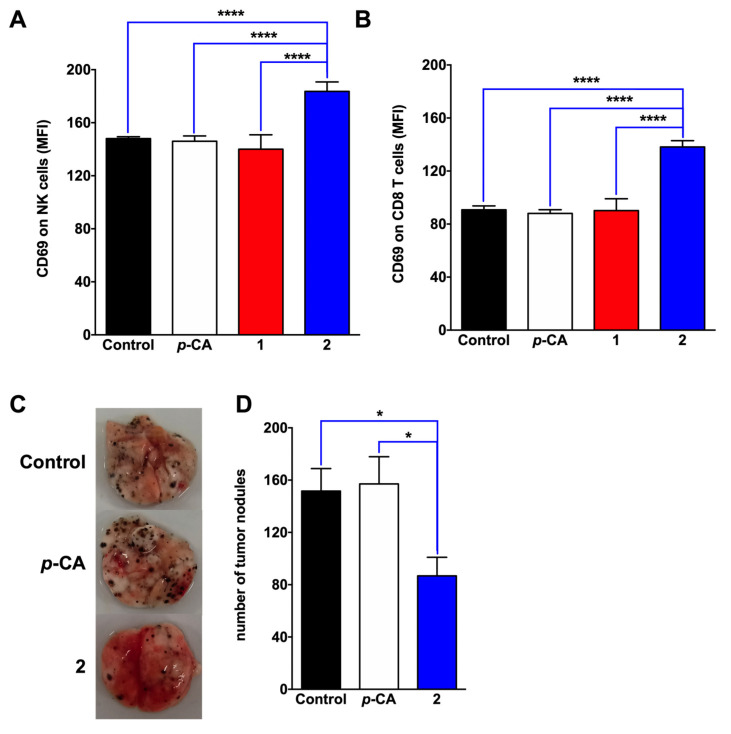
*N*-butyl *p*-coumarate stimulates the NK and CD8 T cell activation and controls the tumor growth. (**A**,**B**) splenocytes from C57BL/6 were incubated with *p*-coumaric acid (*p*-CA, white), ethyl *p*-coumarate (**1**, red) or n-butyl *p*-coumarate (**2**, blue) at 0.1 mM for 6 h and the CD69 expression was analyzed by flow cytometry in the NK (**A**) and CD8 T (**B**) cells. The figure represents mean ± SD of one single experiment (n = 5/group). C-D: C57Bl/6 mice were injected i.p. with a saline solution containing *p*-CA or compound **2** (2 mg/animal) for 5 consecutive days. Control animals received the corresponding DMSO concentration. At day three of the treatment, 2 × 10^5^ B16-F10 melanoma cells were inoculated i.v., and the number of tumor nodules was assessed fifteen days later. (**C**) representative figure from one animal/group. (**D**) The figure represents the mean ± SEM of the lung nodule counts from two individual experiments (n = 4–5/group). * *p* < 0.05. **** *p* < 0.0001.

The stimulation of the naive murine splenocytes with *p*-CA or ester derivatives, reveals that compound **2** induced a significant increase in the expression of CD69, an early activation marker, in the NK cells and the CD8 T lymphocytes, suggesting a non-specific stimulatory effect on these cellular populations (Figure 5A,B, respectively). Next, to determine the impact of this phenomenon on the natural response to the tumor cells, the C57BL/6 mice were treated with *p*-CA or compound **2**, for 5 consecutive days, starting 3 days before the B16-F10 inoculation. Although the *p*-CA treatment failed to control the tumor growth, animals treated with compound **2** presented with lower counts of lung tumor nodules, compared with the control and *p*-CA-treated groups (Figure 5C,D).

## 4. Discussion

Despite all of the efforts to understand the biological pathways that control mutagenesis, cancer treatment remains one of the challenges of modern medicine. Parallel to the increase in life expectancy, the burden of cancer cases reached unprecedented levels, compelling a race for modern, technological, and more personalized therapeutics. Unfortunately, these advances will not reach many cancer patients, mainly due to the imbalance between the high cost of these approaches and the growing social inequality [10,11]. Therefore, the discovery of new drugs to treat and manage cancer must meet the required efficacy criteria and be affordable and accessible for all patients [12]. Nature has always been closely related to pharmacology, by providing drugs used today, such as paclitaxel, or as the starting point to develop safer and more effective synthetic alternatives [13,14]. In this sense, *p*-CA, a phenolic acid found in fruits, vegetables, and herbs, has a great potential due to its antitumor capacity [3,15,16].

Following the identification of a hit, further molecular modifications can improve the potency and safety or modulation of the physicochemical properties that impact its pharmacokinetics. We have previously demonstrated that *p*-coumarate esters were more active than the parent compound in inhibiting the melanin synthesis in both in vitro and ex vivo experiments. This improvement in activity probably results from additional interactions of the alkyl chain in the active site of tyrosinase and the absence of the ionizable carboxylic acid moiety that hamper the permeation through the cell membrane [5]. Despite the antimelanogenic activity, these compounds should also control the melanoma activity, since there is evidence that melanin controls the metastatic behavior of the melanoma cells [17]. Therefore, in the first set of experiments, we aimed to determine the cytotoxic effect of compounds **1** and **2** on the B16-F10 murine melanoma cells.

In accordance with previous studies, the most significant cytotoxic activity of *p*-CA on the B16-F10 cells was achieved when doses higher than 1 mM were employed (data not shown) [3,15]. Thus, 1 mM was the starting concentration chosen to compare the efficacy of the *p*-CA derivatives with the parent acid. Our results show that both *p*-coumarate esters exert a more significant antitumor effect than *p*-CA by the direct cytotoxicity against tumor cells and an antiproliferative effect, strongly suggesting a positive role for the increased lipophilicity given by the alkyl chain and the protection against ionization by the esterification of the carboxylic acid.

The LDH is a stable cytoplasmic enzyme found in all cell types, which is released upon the damage of the plasma membrane, whereas crystal violet stains the adhered live cells. Briefly, the dying cells release LDH and lose their adherence, increasing the LDH in the supernatant and decreasing the crystal violet staining. In this sense, the results obtained in the culture of the B16-F10 cells with compounds **1** and **2** suggested two different phenomena. The inverse relationship between the cell damage (LDH) and the percentage of the live cells indicated a direct cytotoxic effect at higher doses. In contrast, it is possible to observe the dissociation between the LDH levels and the lower percentage of the viable cells at lower doses. Since crystal violet represents live adhered cells, and the B16-F10 cells proliferate spontaneously, we hypothesized that the lower crystal violet staining in the absence of the LDH release, resulted from a decrease in the B16-F10 proliferation rate. Therefore, we used flow cytometry to analyze the proliferative behavior of B16-F10 after incubation with low doses of *p*-CA and its derivatives.

The inhibition of the CFSE decay in B16-F10 over 72 h of culture corroborated the antiproliferative effect of compounds **1** and **2**. Although the mechanisms involved in this process are not understood, we observed that both derivatives induced a cell cycle arrest at the G0/G1 phases and that the compound **1** and **2**-treated cells failed to form colonies in long-term culture conditions. To corroborate this idea, we analyzed the expression of Ki-67, a protein produced during all cell cycle phases, in which the concentration is controlled by the stage-specific regulation of the mRNA transcription and protein degradation, achieving the maximum levels in mitosis and the minimum levels in the late G1 [18,19]. In this sense, the decay in the Ki-67 expression after treatment with compounds **1** and **2**, reflects the protein degradation during the G0/G1 phase of the cell cycle (Figure S4). Similarly, both compounds also exhibited a cytotoxic and antiproliferative effect on the human melanoma cell line, SK-MEL-25. However, the cell cycle arrest observed in this cell line was predominantly at the S phase (compound **1**) or S and G2/M phases (compound **2**). Indeed, contrary to what was observed in the B16-F10 cells, the expression of Ki-67 increases in the first 12 h after treatment with compounds **1** and **2**, indicating the cell arrest in phases S or G2/M (Figure S5).

The cell cycle is the mechanism that drives the cell proliferation in quiescent cells (G0) through four phases: G1, DNA synthesis (S), G2, and mitosis (M). The complexes formed between cyclins and regulatory proteins named cyclin-dependent kinases (CDKs) or CDK inhibitors (CDKI) that, respectively, promote the cell cycle progression or inhibition, tightly regulate this process [20]. In malignant cells, the overexpression of cyclins or the inactivation of CKDIs jeopardizes the cell cycle control, resulting in the uncontrolled cell proliferation. Although discussing the cell cycle arrest differences between B16-F10 and SK-MEL-25, is merely allusive, a similar phenomenon has been previously described. For example, *p*-CA induces the cell cycle arrest of the murine melanoma B16 cell in the G0/G1 phase by modulating the activity of cyclin E-CDK2, whereas, on the human melanoma A375, the modulation of the cyclin A-CDK2 complex drives the cell cycle arrest in the S phase [3]. In the human colon cancer CaCo-2 cell line, *p*-CA up-regulates the CDK inhibitor 1 (CDKN1A) expression and down-regulates other cell cycle-promoting proteins, resulting in the cell cycle arrest at the G2/M phase [21]. Thus, compounds **1** and **2**, likewise *p*-CA, modulate different pathways of the cell cycle inhibition that may vary according to the tumor cell type.

The improved antitumor capacity of the *p*-coumarate derivatives probably relies on two complementary phenomena. Since *p*-CA is an acidic compound, it is predominantly ionized in the physiological pH, which hampers its ability to penetrate through the cellular membrane. In contrast, the esters are neutral compounds under physiological conditions and present a better penetration capacity. Once these compounds achieve the cytoplasm, the natural cell esterases hydrolyze them to *p*-CA, acting as bioprecursors of *p*-CA. Our previous results support this hypothesis, where their antimelanogenic effect is correlated with their lipophilicity [5]. Although the hydrophobic interactions with the putative molecular target can also play a role in this effect, a higher cell penetration can also explain the higher potency of compound **2,** compared to **1**.

The effect of *p*-CA on the immune responses remains a controversial subject. Although *p*-CA exerts a potent immunosuppressive impact on autoimmune inflammatory diseases, such as rheumatoid arthritis, it also seems to stimulate the activity of NK and cytotoxic T lymphocytes [22,23]. Therefore, we stimulated total splenocytes with *p*-CA and its derivatives. Although no effect was observed after the incubation with *p*-CA or compound **1**, a significant increase in the expression of CD69 in the CD8 T lymphocytes and NK cells occurred upon the stimulation with compound **2**. The CD69 is well recognized as an early activation marker for hematopoietic cells induced upon several stimuli and with different biological functions, including home addressing and cytolytic activity, indicating a stimulatory activity of compound **2** on immune cells [24,25]. Because we used naive splenocytes, this phenomenon probably reflects a non-specific stimulatory effect on immune cells, due to its chemical modification.

Finally, since compound **2** exerted a stimulatory effect on the NK and CD8 T cells, two immune cell populations involved in the natural response against tumors, we sought to determine its in vivo antitumor potential against the B16-F10 melanoma cell. Our findings showed that the treatment with compound **2** reduced the number of tumor nodules in the lungs, compared to the non-treated or *p*-CA-treaded group. Although the mechanisms driving this antitumor effect remain unclear, it is reasonable to suppose that it results from the synergism between the direct cytotoxicity on the melanoma cells and the stimulation of the immune cells responsible for the natural response against the tumor.

## 5. Conclusions

The results of this work show that the modifications on the carboxylic acid moiety of *p*-CA to generate more lipophilic and neutral ester analogs positively contribute to the gain in the cytotoxic and antiproliferative activity against the murine and human cancer cell lines, conferring to these compounds a unique antitumor potential. Moreover, the more lipophilic compound **2** is highlighted as a promising antitumor agent and a potential prototype for further modification to achieve more active compounds.

## Data Availability

All data are available under reasonable request.

## Supplementary Materials

The following supporting information can be downloaded at: https://www.mdpi.com/article/10.3390/biomedicines11010196/s1, Figure S1: Chemical structure of *p*-CA and its ethyl (**1**) and butyl (**2**) analogues; Figure S2: Ethyl *p*-coumarate (**1**) and *n*-butyl *p*-coumarate (**2**) exert a more cytotoxic effect on the B16-F10 cells, compared to *p*-coumaric acid (*p*-CA); Figure S3: n-butyl *p*-coumarate (**2**) exerts a more cytotoxic effect on the SK-MEL-25 cells, compared to *p*-coumaric acid (*p*-CA) and ethyl *p*-coumarate (**1**); Figure S4: Ethyl *p*-coumarate (**1**) and n-Butyl *p*-coumarate (**2**) inhibits the accumulation of the Ki-67 in B16-F10 cells; Figure S5: Ethyl *p*-coumarate (**1**) and n-butyl *p*-coumarate (**2**) promotes the early accumulation of the Ki-67 in SK-MEL-25 cells.
