# Supplementary figures and images for: Esterification of p-Coumaric Acid Improves the Control over Melanoma Cell Growth
Source: Biomedicines. 2023 Jan 12;11(1):196. doi: 10.3390/biomedicines11010196 (PMC9855326; doi:10.3390/biomedicines11010196)

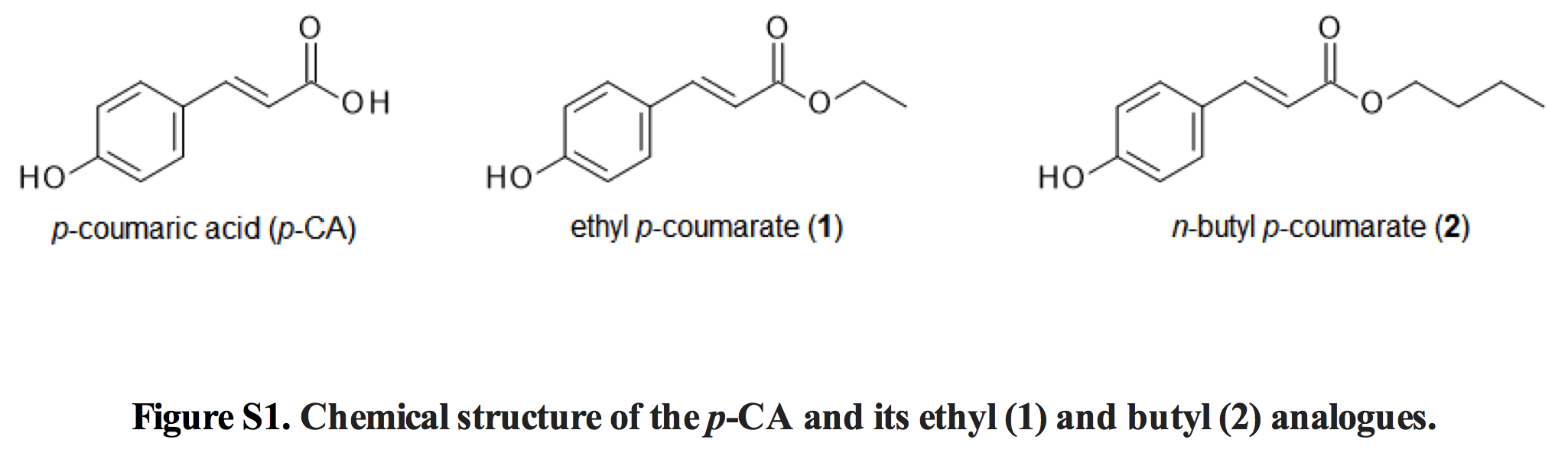

Supplement: Supplementary file 1 [file biomedicines-11-00196-s001.zip › Figure S1.tiff]

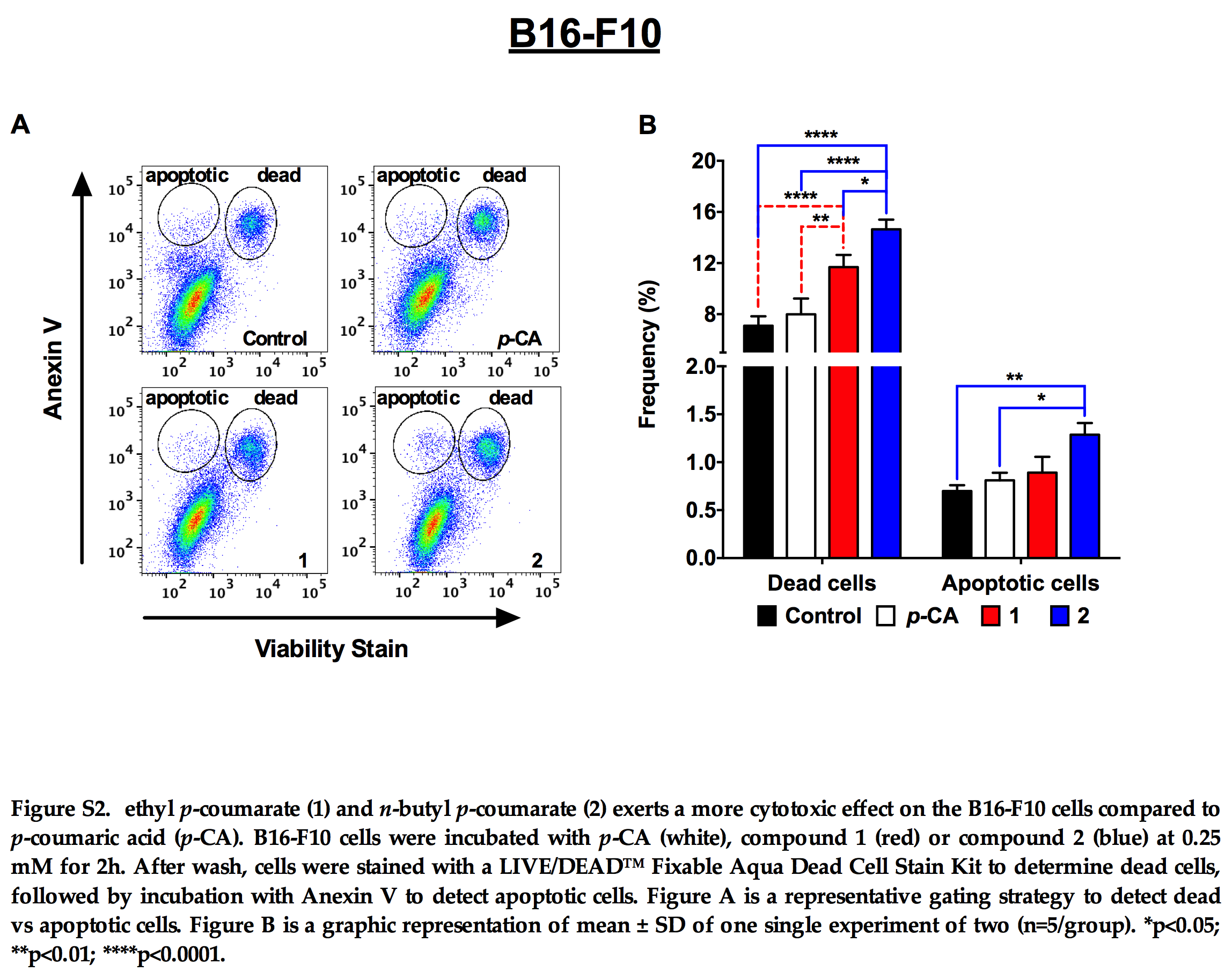

Supplement: Supplementary file 1 [file biomedicines-11-00196-s001.zip › Figure S2.tiff]

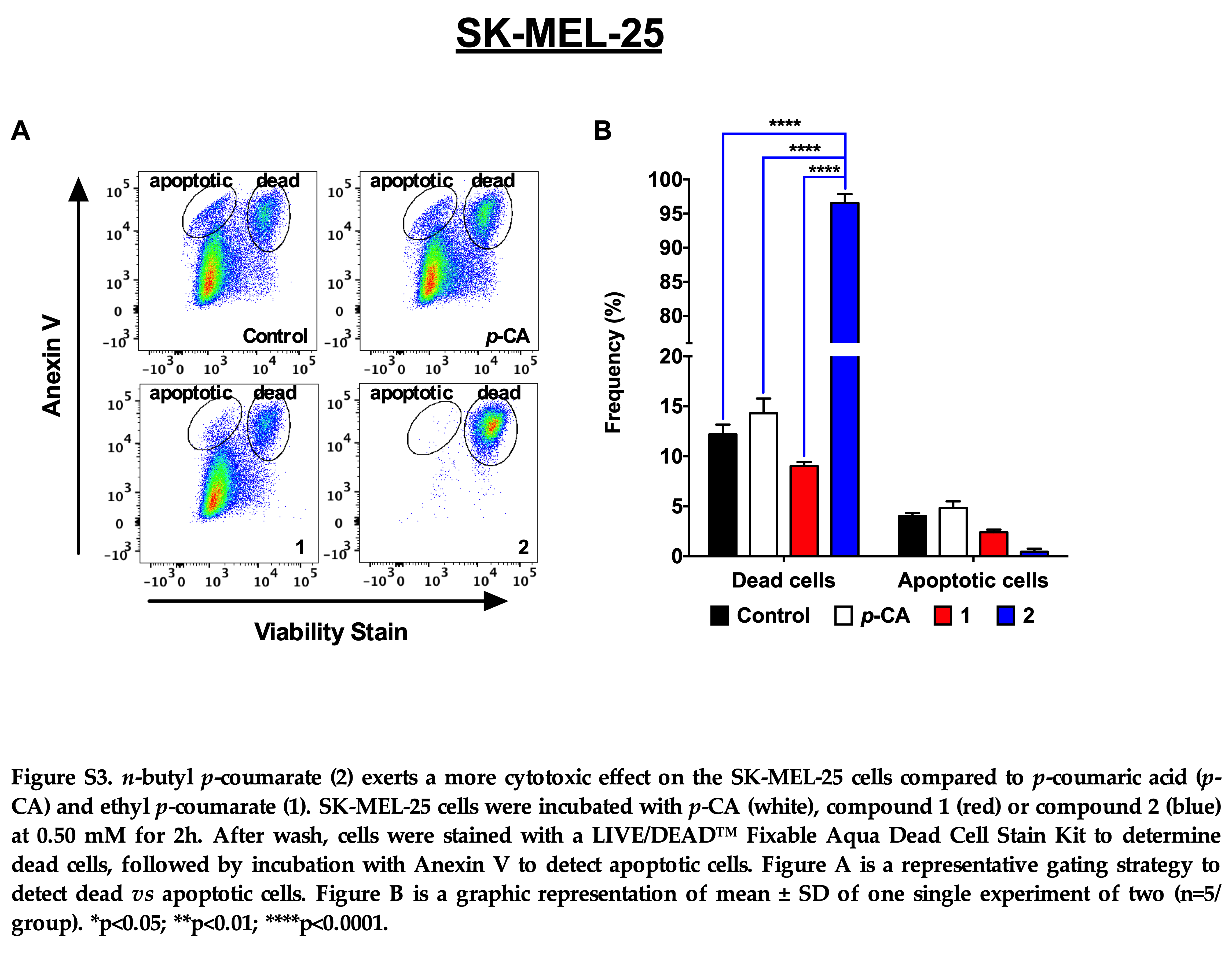

Supplement: Supplementary file 1 [file biomedicines-11-00196-s001.zip › Figure S3.tiff]

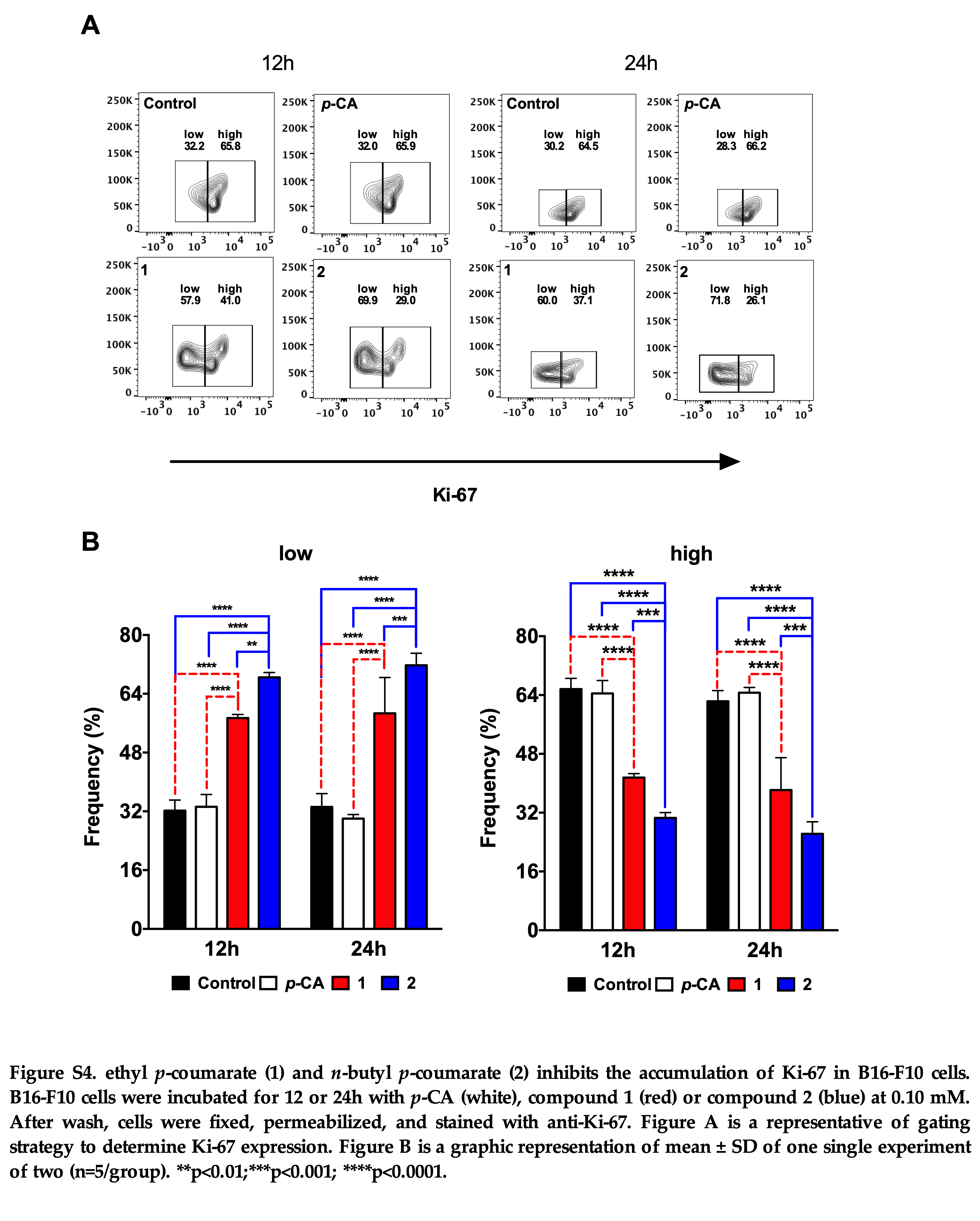

Supplement: Supplementary file 1 [file biomedicines-11-00196-s001.zip › Figure S4.tiff]

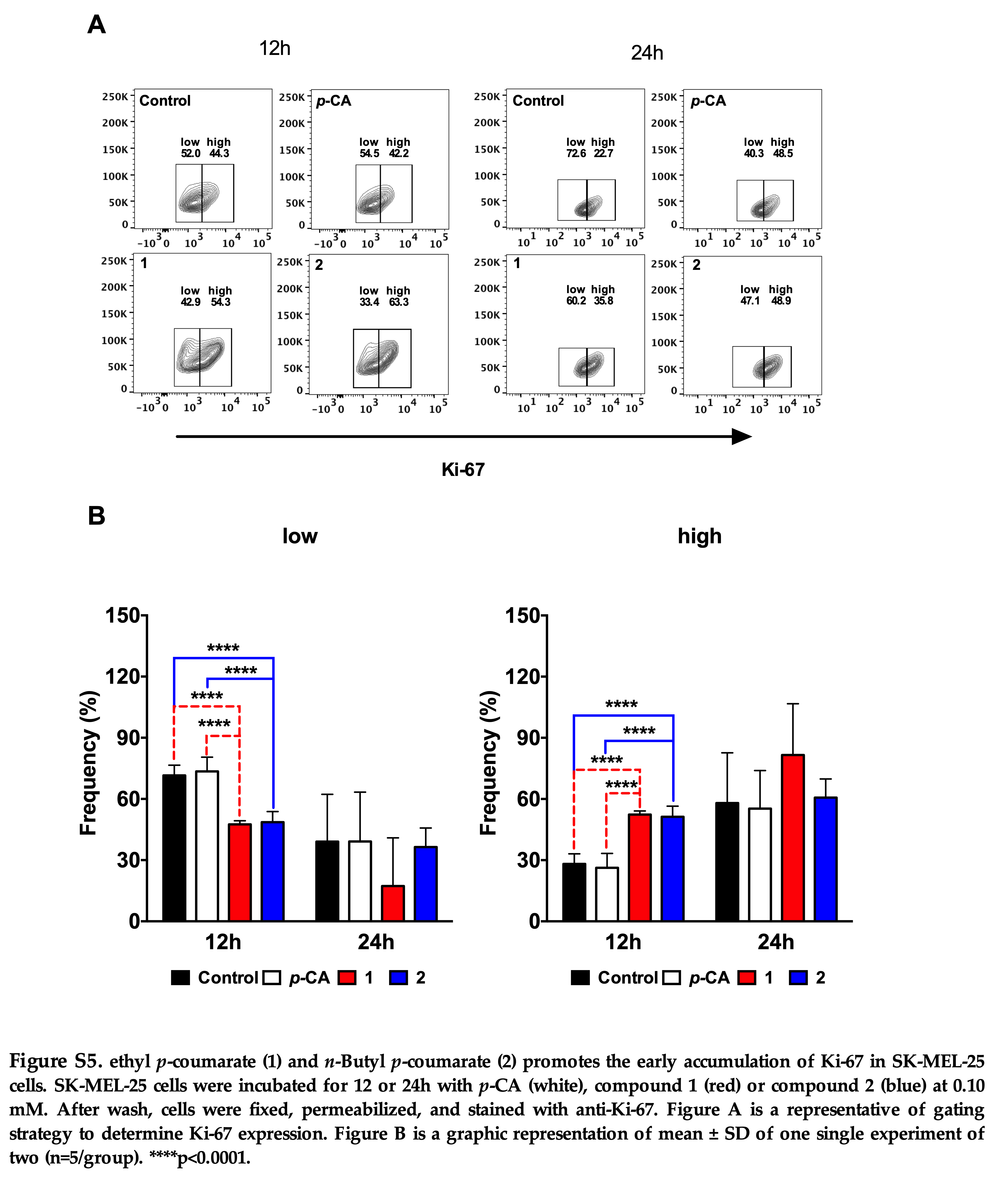

Supplement: Supplementary file 1 [file biomedicines-11-00196-s001.zip › Figure S5.tiff]
